# Editorial of Special Issue “Adipokines 2.0”

**DOI:** 10.3390/ijms21030849

**Published:** 2020-01-28

**Authors:** Christa Buechler

**Affiliations:** Department of Internal Medicine I, Regensburg University Hospital, 93053 Regensburg, Germany; christa.buechler@klinik.uni-regensburg.de; Tel.: +49-0941-944-7009

## Abstract

This editorial aims to summarize the 19 scientific papers that contributed to the Special Issue “Adipokines 2”.

The Special Issue, “Adipokines 2.0” of the International Journal of Molecular Sciences is a follow up of the Special Issue “Adipokines” published two years ago. 

Proteins secreted from fat tissues are collectively referred to as adipokines. Circulating levels of these proteins are—with a few exceptions—increased in obesity. Hundreds of adipokines were discovered and annexins, well known as regulators of membrane-related events such as exocytosis, may affect adipokine release from fat tissues [1]. 

Adipokines promote metabolic and cardiovascular diseases and are biomarkers for obesity-associated comorbidities. Metabolically healthy obesity is associated with an adipokinome similar to that of normal weight mice, providing more evidence for a central role of adipose tissue produced proteins in metabolic diseases [2]. Weight loss improves the parameters of insulin sensitivity and hypertension, but most patients regain weight. Adipocytes cultivated in medium with low, and later on high glucose, showed a different secretome in comparison to the control cells. These differentially released proteins may contribute to worse metabolic parameters in obese patients after weight regain [3]. 

Physical activity is linked to improved metabolic health in normal weight and obesity. Adipo-myokines are proteins secreted by adipose tissues and skeletal muscle and seem to have a role herein. These molecules may be useful to classify different types of obesity and develop individualized therapeutic strategies [4]. 

Excessive gestational weight gain increases the risk of neonatal and maternal complications. The adipokine secreted frizzled-related protein 5 (SFRP5) improves metabolic function. Serum as well as umbilical cord blood levels were low in women with an extreme increase in weight during pregnancy [5]. Leptin was higher in the serum and cord blood of male infants born from mothers with excessive gestational weight gain. Such an induction was not observed for female babies [6]. Adipokine levels correlated with neonatal anthropometric measurements and may contribute to greater risk of obesity and metabolic disease in later life [5,6].

Excess body weight is a risk factor for insulin resistance, hypertension, and cardiovascular diseases. Epicardial adipose tissue contributes to cardiac enlargement in the obese and this involves deregulation of prostaglandin E2 [7]. Overweight and obesity are, moreover, linked to a higher risk of different cancers [8,9]. This was studied in detail for the adipokine chemerin. Of note, chemerin was shown to impair and to improve insulin sensitivity, to have pro- and anti-inflammatory activities and to exert pro- and anticancer effects [8,9,10]. These antagonistic effects may be in part attributed to the different biologic activities of the chemerin isoforms [8]. The role of chemerin in cancer diseases was nicely summarized in two review articles [9,10]. An original investigation tested whether chemerin may serve as a biomarker to discriminate patients with primary and secondary liver tumors. This study identified an association of serum chemerin with hypertension and hypercholesterolemia in the tumor patients [11]. Serum chemerin could, however, not distinguish patients with hepatocellular carcinoma and colorectal liver metastases [11]. 

Adipokines are involved in the pathogenesis of reproductive disorders. The roles of chemerin, visfatin, resistin, and apelin in fertility and associated diseases were summarized in a review article with various informative and clear illustrations [12].

A potential role of chemerin in reproductive function was supported by the finding that plasma levels as well as the expression of its receptors, chemokine-like receptor 1, G protein-coupled receptor 1, and C-C motif chemokine receptor-like 2 fluctuated throughout the estrous cycle and pregnancy in the porcine hypothalamus [13]. 

Other approaches have been to determine the release of adipokines by tissues other than fat. One study analyzed the levels of chemerin, apelin, and omentin in follicular fluid and ovarian granulosa cells. Study cohorts were women with polycystic ovary syndrome, women with a polycystic ovary morphology, and the controls. Differential abundance of these adipokines in the patients suggested a possible role in the pathophysiology of polycystic ovary syndrome [14].

Adipokines are expressed by different cells in the joint microenvironment. Locally produced as well as systemic adipokines contribute to osteoarthritis and rheumatoid arthritis. Different studies have analyzed the pathophysiological role of various adipokines (e.g., adiponectin, leptin and so on). These studies were nicely summarized in a review article [15]. Interestingly, the serum levels of most of these adipokines were higher in the patients [15]. A separate study investigated the effect of treatment with the anti-interleukin-6 receptor antibody tocilizumab in patients with rheumatoid arthritis. Four months of therapy was associated with higher resistin levels and lower adiponectin whereas leptin was not altered [16]. After treatment, adiponectin and resistin serum concentrations were similar to the controls, suggesting the normalization of these parameters [16]. 

Moreover, adipokines were also analyzed as potential biomarkers in sepsis and critical illness. Here, prospective studies are required to finally evaluate the prognostic relevance of the different proteins measured. The heterogeneity of these patient cohorts may limit the diagnostic potential of circulating adipokine levels [17]. 

The role of adipose tissue in the pathophysiology of most diseases is greatly unknown. There is mounting evidence though that adipokines act in the brain, and the role of leptin in the control of food consumption has been well described. Adiponectin protected mice from high fat diet induced hypothalamic inflammation [18]. Microglia, as the resident macrophages of the central nervous system, expressed both adiponectin receptors and were essential for the protective activity of adiponectin [18].

The neuroprotective effects of the adipokines leptin, adiponectin, chemerin, apelin, and visfatin indicate a potential role for these molecules as therapeutic targets in neurodegenerative diseases [19].

Overall, these 19 contributions published in this Special Issue further strengthen the essential function of adipokines in health and in various diseases. Different adipose tissue depots may have specific functions and detailed analysis of their secretome may provide more insight into the connection between fad pads and physiology.
